# Purification, Characterization and anticancer activity of L-methionine γ-lyase from thermo-tolerant *Aspergillus fumigatus*

**DOI:** 10.1186/s12934-023-02019-z

**Published:** 2023-01-12

**Authors:** Mahmoud H. Hendy, Amr H. Hashem, Waleed B. Sulieman, Mahmoud H. Sultan, Mohamed Abdelraof

**Affiliations:** 1 Botany and Microbiology Department, Faculty of Science, Al-Azhar University, Cairo, 11884 Egypt; 2 Microbial Chemistry Department, National Research Centre, Dokki, Cairo, 12622 Egypt

**Keywords:** L-methionine γ-lyase (MGL), *Aspergillus fumigatus*, Purification, Characterization, Anticancer activity

## Abstract

Purification of L-methionine γ-lyase (MGL) from *A. fumigatus* was sequentially conducted using heat treatment and gel filtration, resulting in 3.04 of purification fold and 73.9% of enzymatic recovery. The molecular mass of the purified MGL was approximately apparent at 46 KDa based on SDS-PAGE analysis. The enzymatic biochemical properties showed a maximum activity at pH 7 and exhibited plausible stability within pH range 5.0–7.5; meanwhile the highest catalytic activity of MGL was observed at 30–40 °C and the enzymatic stability was noted up to 40 °C. The enzyme molecule was significantly inhibited in the presence of Cu^2+^, Cd^2+^, Li^2+^, Mn^2+^, Hg^2+^, sodium azide, iodoacetate, and mercaptoethanol. Moreover, MGL displayed a maximum activity toward the following substrates, L-methionine < DL-methionine < Ethionine < Cysteine. Kinetic studies of MGL for L-methioninase showed catalytic activity at 20.608 mM and 12.34568 µM.min^−1^. Furthermore, MGL exhibited anticancer activity against cancerous cell lines, where IC_50_ were 243 ± 4.87 µg/ml (0.486 U/ml), and 726 ± 29.31 µg/ml (1.452 U/ml) against Hep-G2, and HCT116 respectively. In conclusion, *A. fumigatus* MGL had good catalytic properties along with significantly anticancer activity at low concentration which makes it a probably candidate to apply in the enzymotherapy field.

## Introduction

Thermophilic and thermotolerent fungi are wildly used to produce novel and thermostable enzymes as proteases, cellulases, xylanases and methionase [1]. *Aspergillus* species have been reported to produce extracellular MGL [2]. Abu-Tahon, Isaac [2] reported that *A. ustus* has ability to produce L- methionase using sub-merged and solid state fermentation. El-Sayed [3] used *A. flviceps* for extracellular production of L- methionase using solid state fermentation. Sulfur-containing amino acids (SAAs) are important components in a variety of natural processes and are found in all living things. Environmental changes control the biosynthetic and catabolic pathways of SAAs, which are so differ between organisms and developmental stages [4, 5]. One of the key enzymes that responsible for the SAAs release to the environment is a pyridoxal phosphate (PLP)-dependent hydrolytic enzyme L-methionine-γ-lyase (MGL) (EC 4.4.1.11; MGL). The major substrate for MGL is L-methionine, which was decomposed via the γ-elimination mechanism to resulting α-ketobutyrate, methanethiol (MTL), and ammonia, as well as it was degraded L-cysteine via the β-elimination, and α, β-replacement of S-substituted [6, 7]. Microbial MGL has been attention paid by many authors, which was produced by different microorganisms including bacteria, filamentous fungi, and yeast [8–10]. Several wild-type fungal strains were extensively studied for the production, and purification of MGL [1, 11, 12]. However, MGL was not reported from the fungal that isolated from the extreme habitats yet. In which, these studies would be worthy to open a new way to discovering a novel properties for the fungal MGL. Besides, one of the most applications of fungal MGL is related with a reduced immunogenic response during tumor therapy, which may be linked to the structural competency of the humannimmune system [13]. MGL is a strong anticancer agent that has been demonstrated to active against a variety of cancer cell lines such as, kidney, breast, lung, colon, and glioblastoma [14]. In a woman with high-stage ovarian cancer, MGL therapy resulted in a 50% drop in circulating methionine and a 70% reduction in prostate-specific antigen [15]. Recent major efforts in cancer studies focus on designing new forms of chemotherapy that utilize the clear differentiation between cancer and normal cells like that used in case of MGL [6, 16]. Methionine dependency is a distinct characteristic of certain types of cancer cells [6]. Selectively, cancer cells were depending on methionine for their proliferation and the depriving these cells of methionine by MGL caused prevent cancer cells to growth, cancer cells have not any mechanism to substitute this loss of methionine. Thus, enzymotherapy was considered a potent way to killing the cancer cells by MGL, since the dependency of cancer cells to methionine is due to enhanced requirements of methionine for regulation of DNA expression in cancer cells and high protein synthesis [8]. Additionally, neither healthy volunteers nor cancer patients experienced any negative side effects from the oral administration of MGL [15]. Herein, this work was designed to purify MGL which produced from the thermo-tolerant *A. fumigatus* and investigates its biochemical characteristics. Furthermore, to assess the anticancer efficacy of the purified enzyme against various cancer cell lines in compared to normal cell line.

## Materials and methods

### Materials

The chemicals and reagents used in this study came from the following sources: L-methionine derived from (Merk, Germany). Sodium methanethiolate was purchased from Sigma-Aldrich, Commassi Brilliant Blue G-250, 5,5-Dithiobis-2-nitrobenzoicacid (DTNB), pyridoxal-5-phosphate (PLP), and bovine serum albumin (BSA) (Sigma, St. Louis, USA). Pharmacia Biotechnology provided Sephadex G-100 and DEAE-cellulose (Sweden). Potato dextrose agar (PDA) (Conda lab, Spain). The American Type Culture Collection provided cancer and normal cells for this study (ATCC, Rockville, MD, USA). The rest of the compounds are of the highest analytical quality.

### Methods

#### Thermo-tolerant fungi strain and production conditions

In our previous study, *A. fumigatus* was isolated from Egyptian soil and selected as the most effective producer of MGL [1]. The MGL production medium used is a modified Basal medium, contained (g/L): L-methionine, 3; Polypeptone, 1; Glycerol, 1; K_2_HPO_4_, 1; KH_2_PO_4_, 1; MgSO_4_.7H_2_O, 1 and yeast extract, 0.25. In addition, pH of growth medium was adjusted to 7 using potassium phosphate buffer 0.075 M. Cells were extracted by centrifugation (5500 rpm, Herml, Germany) after 4 days of shaking (150 rbm, New Brunswick, USA). As a crude enzyme, the clear supernatant was employed [13].

#### L-methioninase assay procedure

MGL activity measurement was assessed based on the demethiolation reaction according to method used by Selim et al. [14]. In this regard, 20 mM L-methionine in 0.05 M potassium phosphate buffer pH 7.0, 0.01 mM pyridoxal phosphate (PLP), 0.25 mM DTNB, and the filtrate in a final volume of 1 ml were used in the assay process. Controls without filtrate and heat denatured filtrate (at 60 °C for 10 min) were made separately after 1 h of incubation at 30 °C. Methanethiol (MTL) was released from L-methionine and spontaneously reacted with 5,5 di-thio-bis-2-nitrobenzoicacid (DTNB), yielding a yellow-colored product (thionitrobenzoic acid) can detected spectrophotometrically at 412 nm absorbance. Sodium methanethiolate as a standard reference was used for measuring emitted amount of MTL. Under optimized assay, one unit (U) of MGL known as the amount of MGL that releases 1µmole of methanethiol per minute.

#### Protein determination

Protein concentration was determined according to the method of Bradford [17] with bovine serum albumin as standard.

#### Purification of *A. fumigatus* L-methioninase

Purification of *A. fumigatus* MGL was established according to previous scheme used by Selim et al. [18].

#### Determination of purity and molecular mass of *A. fumigatus* L-methioninase

Checked of the *A. fumigatus* L-methioninase purity was implemented using polyacrylamideegel electrophoresisn(SDS-PAGE) procedure as described by LAEMMLI [19] using standard marker proteins. Besides, applied of UV–visible scan for the purified enzyme can also be referred to the purity of MGL [6].

#### Biochemical characterizations of *A. fumigatus* L-methioninase

##### Effect of pH on the enzyme activity and stability

The effect of pH value on pure enzyme activity was determine using 0.05 M of different buffers (i.e.) acetate (pH 4.0- 6.0); potassium phosphate (pH 6.5- 8.0) and carbonate buffer (pH 9). By incubation with different buffers solutions (4–10.5), each reaction enzymatic activity was estimated. To determine its pH stability of the enzyme was preincubating the enzyme solution at different pH values ranging from 4 to 10.5 for 1 h at room temperature 33 °C in the absence of substrate. After the pre-incubation period, the pH of the enzyme solution was adjusted to pH 7, which is the optimum pH for the standard assay method, and the residual activities were measured using the standard method.

##### Effect of temperature on the enzyme activity and stability

Incubating the reaction mixture in 50 mM potassium phosphate buffer (pH 7) at several temperatures (30–80 °C) for 20 min for the optimal temperature on pure enzyme activity was performed. The percentage of peak enzyme activity was used to calculate the residual enzyme activity. In addition, thermal stability assays were carried out by pre-incubating the purified enzyme at several temperatures ranging (40–90 °C) for 100 min.

##### Effect of some metal ions and chemical reagents on the enzyme activity

MGL was preincubated with different metal ions (i.e. Ca^2+^, K^+^, Na^+^, Ni^2+^, Mg^2+^, Co^2+^, Cr^2+^, Cd^2+^, Cu^2+^, Zn^2+^, Fe^+2^and Ba^+^) and some chemical reagents included SDS, EDTA and SH group (i.e. DTT and β-Mercaptoethanol) for 4 h at room temperature to investigate their inhibition or activation effects. Following that, the enzyme solution was added to its substrate and the residual activity was determined by the standard assay method.

##### Determination of kinetic parameters

Purified Lmethioninase was utilised in this experiment to estimate kinetic parameters such as MichalisMenten constant (K_m_) and maximum velocity (V_max_) by incubating the enzyme with various concentrations of each substrate (20, 40, 80, and 160 mM) under optimal assay conditions. A Lineweaver–Burk plot was used to calculate the apparent K_m_ and V_max_ of pure enzyme.

#### In-Vitro anticancer activity assay

Anticancer activity of Purified L-methioninase was investigated against liver HepG2 and colon HCT116 cancer cell lines, as well as a normal cell line (humannnormal melanocyte, HFB4) combared to doxorubicin. The cells were cultivated and maintained in RPMI-1640 mediaawith 10% heattinactivated fetal calf serum (GIBCO), 100 U/ml penicillin, and 100g/ml streptomycin. The cells were kept at 37 °C in aahumidified environment containing 5% CO_2_. The cancer cell lines were suspended in medium at cells concentration of 5 x 104 cell/well in Corning^®^96-well tissue culture plates grown in a 25 cm 2 flask in 5 ml of culture medium, and then incubated for 24 h. Then, the tested enzyme was added into 96 well plates (six replicates) to achieve eight concentrations of enzyme. Six vehicle controls with media orr0.5% DMSO were ran for each 96ewell plate as aacontrol. After incubationffor 24 h, numbers of viable cells were determined by the MTT test [20–22]. The viability and inhibition percentages were calculated according to these equations:

$$\mathrm{Viability \%}= \frac{\mathrm{Sample\, OD}}{\mathrm{Control\, OD}} \times100$$

$$\mathrm{Inhibition \%}=100-\mathrm{Viability \%}$$

#### Statistical analysis

All Experiments were carried out in triplicate and their results are presented as mean ± standard deviations (SD).

## Results and discussion

### Purification of *A. fumigatus* L-methioninase

Production of *A. fumigatus* L-methioninase was conducted under culture medium that optimized as ascribed of our previous study [1]. Harvesting of the supernatant was performed using cooling centrifuge (10,000 × g for 20 min.) in order to separating the active extracellular crude L-methioninase from the fungal mycelium. Then the obtaining crude enzyme with 12.24 U/mL of activity and protein content with 0.91 mg/mL was purified to apparent homogeneity based on a smaller number of purification steps, involving heat treatment, and Sephadex G100 column [6, 18]. Heat treatment of the protein sample was implemented to removing the denatured protein from the supernatant, a slight decrease in the enzyme activity was noted (11.16 U/mL) and near to 45% reduced in the protein content was observed. The purification fold was reached to 1.76-fold with 23.74 U/mg of specific activity and 81.2% of recovery yield. The active L-methioninase was concentrated under lyophilization process and applied onto Sephadex G100 column. The enzymatic activity was detected in the fractions from 14 to 16 along with high protein content, collected of the active fractions together was carried out with further increased of each of specific activity ( 41 U/mg), and purification fold with 3.04. Successfully purification scheme was obtained with minimum steps with a good enzymatic recovery of 73.96% being valuable for the industry in terms of economic point of view [23]. *A. fumigatus* L-methioninase was subjected under only two purification steps; which was better than those obtained for L-methioninase purification by *Trichoderma harzianum* [12], *A. flavipes* [24], *Candida tropicalis* [14], requires to three purification steps. As can be seen in the purification profile (Table 1), the heat treatment playing an important role, which reducing the undesirable protein with 45%. Utilization of heat treatment at 60 °C was applied for *Citrobacter freundii* L-methioninase purification with 21% yield followed by separation on DEAE-cellulose column only [25]. In addition, heat treatment of L-methioninase from *Streptomyces* DMMHM4 was enhanced the purity by removed more than 50% of protein content [18].

**Table 1:** Purification profile of *A. fumigatus* L-methioninase

Sample	Total protein (mg/ml)	Total activity (U/ml)	Specific activity (U/mg)	Enzyme recovery (%)	Purification fold
Crud L-methioninase	0.91	12.24	13.45	100	1
Heated treatment	0.47	11.16	23.74	90.43	1.76
Sephadex G100 (Gel filtration)	0.22	9.02	41	73.96	3.04

**Fig. 1:**
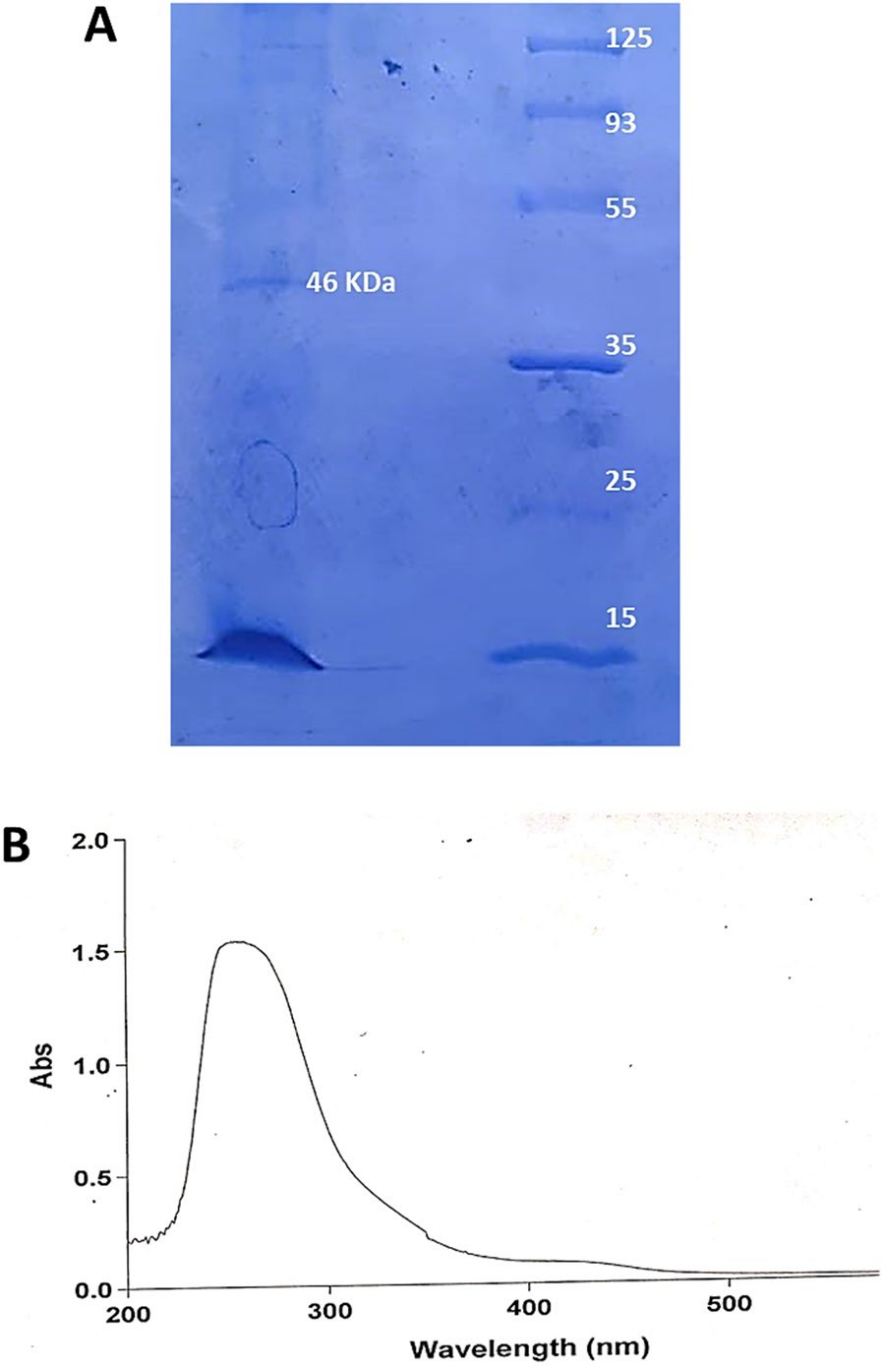
SDS-PAGE (**A**) and UV-Scan of the *A. fumigatus* L-methioninase (**B**)

In order to confirm the enzyme purity and its molecular mass, SDS-PAGE was applied after concentrated the active fractions of Sephadex G100, which demonstrated a single band at approximate molecular mass of 46 kDa with two identical subunits revealed the dimer structure of *A. fumigatus* L-methioninase. Besides, the purified enzyme under UV–visible scanning (Fig. 1) showed two maximum peaks at 280 nm referring its containing aromatic amino acids, and at 420 nm indicating the PLP-enzyme binding domain via aldimine linkage between the aldehyde group of the pyridoxal phosphate and the lysine amino group of the enzyme [24], 6. In accordance with our results, the molecular weight of L-methioninase purified from the fungal sources could range between 43 and 48 kDa, which reported as 47KDa for *A. flavipes* [24]**,** 48 KDa for *T. harzianum* [12], 46KDa for *A. ustus* [2], and 46 KDa for *C. tropicalis* [14].

### Biochemical properties *A. fumigatus* L-methioninase

**Fig. 2:**
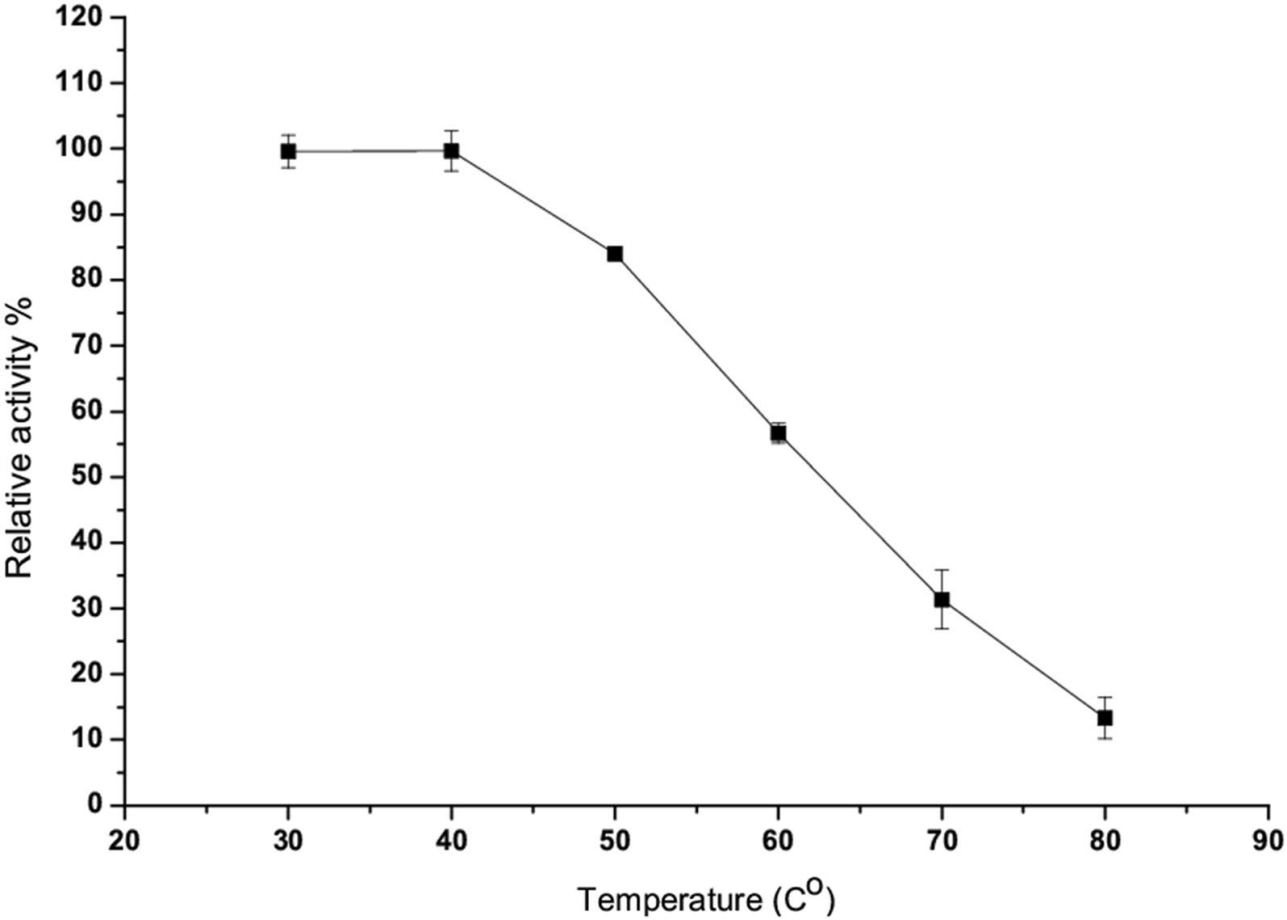
Effect of temperature on *A. fumigatus* L-methioninase

The optimum temperature for L-methioninase activity was determined by incubating the purified enzyme with the standard substrate (L-methionine) at several reaction temperatures (30–80 °C). The temperature profile of *A. fumigatus* L-methioninase was proved to be the most significant L-methioninase activity was found between 30 and 40 °C (Fig. 2). Moreover, as the temperature increases above 40 °C a considerable decreasing in the enzymatic activity was observed. A slight decrease is obtained at 50 °C, while a remarkable enzymatic inactivity was determined over rang (60–80 °C), more than 90% reducing in the original activity was noted at 80 °C.

*A. fumigatus* L-methioninase activity was observed below 40 °C closed to that reported for the purified L-methioninase from *A. flavipes* [24], *T. harzianum* [12], *A. ustus* [2] and *Aspergillus* spp. [26] which was exhibited maximum activity at 35 °C followed by a gradual decrease of its original activity at higher temperatures.

**Fig. 3:**
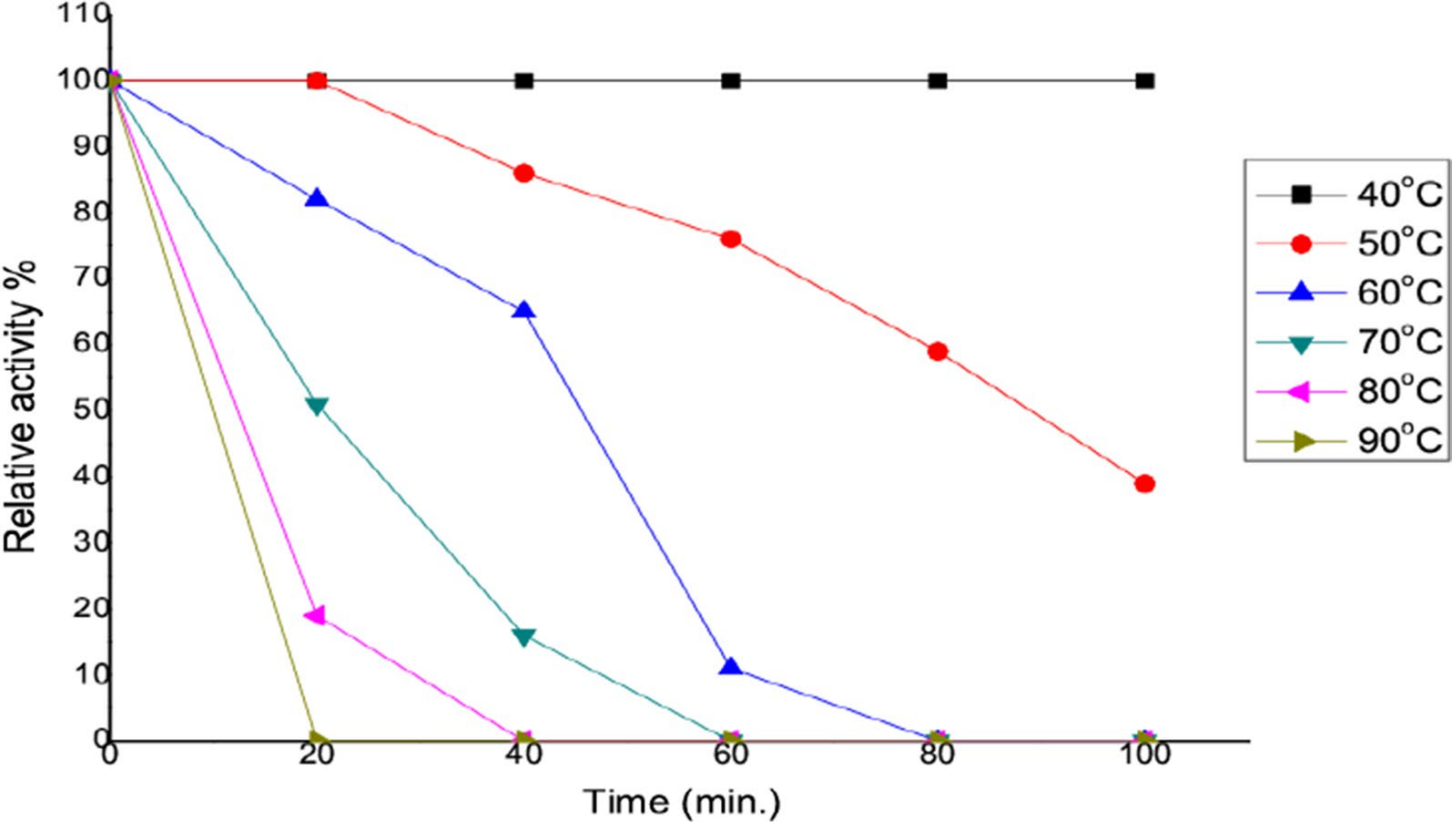
Thermal stability of *A. fumigatus* L-methioninase

On the other hand, the thermal stability of *A. fumigatus* L-methioninase was also evaluated by exposure the purified enzyme to different temperature during 100 min. As shown in (Fig. 3), it could be noticed that, the pure enzyme could stable up to 40 °C for 100 min, which not loss its original activity followed by a slight decrease of the enzyme stability after 40 min. at 50 °C was observed. Furthermore, after heating the purified enzyme at 60 °C for 20 min, the enzyme still retained about 82% of its initial activity. In addition, a severe inhibition of enzyme activity (< 15%) was noticed when the enzyme was incubated at 70 °C, and 80 °C for 20 and 40 min., respectively. In consistence with our results, [12] found that, the purified enzyme from *C. tropicalis* was stable after heating at 45–55 °C for 20 min. In addition, the thermal stability of L-methioninase from *A. flavipes* [24], *A. ustus* [2], and *T. harzianum* [12] displayed a relative catalytic stability below a temperature of 40 °C.

**Fig. 4:**
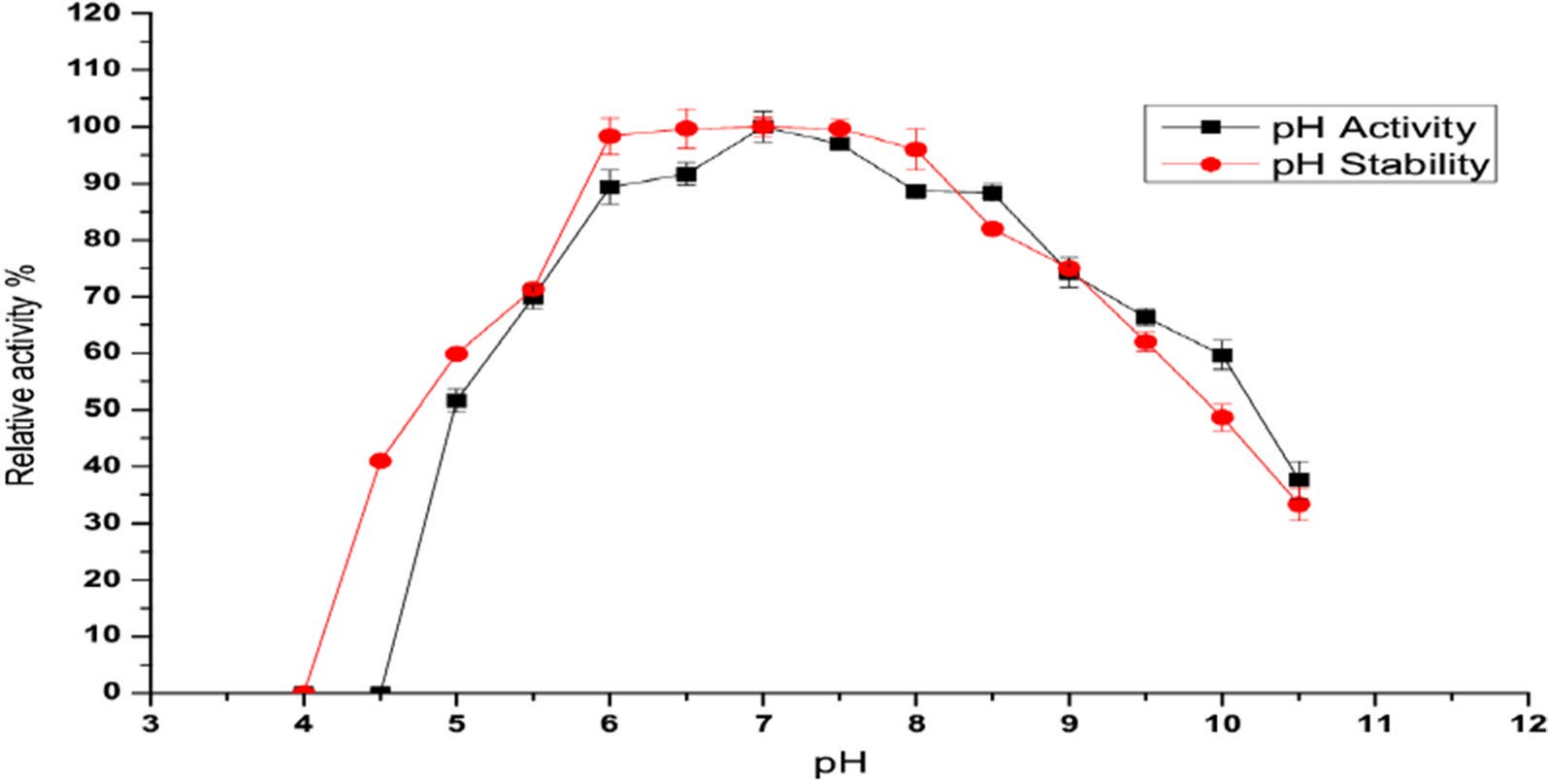
Effect of pH on *A. fumigatus* L-methioninase activity and stability

The effect of pH on *A. fumigatus* L-methioninase activity was examined using different buffer solutions (4–10.5). As shown in (Fig. 4), the optimum L-methioninase activity was obtained at pH 7 adjusted with potassium phosphate buffer. It is clear that, the enzyme activity was increased with the increase of pH values towards the neutrality. Increasing pH values towards alkalinity was accompanied by a slight decrease in enzymatic activity. Moreover, the enzymatic activity rate was remarkable too decreasing when nearing to more acidic pH values. Since, at pH 5.0 the enzyme retained over 51% of its activity, while at pH 4.0 a complete decrease in enzyme activity was observed. In this concern, *A. fumigatus* L-methioninase had maximum activity in the neutral pH values and gradually decrease at lower or higher pH values was detected, which may be due to the profoundly effect of the ionic strength on amino acid or carboxylic groups which could leading to markedly affected the catalytic site and conformation of enzymatic structure [6, 24]. The significant decrease in the enzyme activity below or above the optimum may also be due to the lowered affinity between the enzyme and its substrate In addition, In agreement with our results, neutral to slightly alkaline pH values were reported for optimum activity of L-methioninase purified from *A. flavipes* [24]. Selim et al. [14] reported the optimum pH value for *C. tropicalis* L-methioninase to be 6.5. *T. harzianum* L-methioninase displayed broad activity in the pH range of 5 to10 with maximum activity at pH 8 [12]. Moreover, *A. ustus* L-methioninase exhibited maximum activity in the alkaline pH range at 8.5 [2].

L-methioninase stability profile under different pH buffers was summarized in (Fig. 4), significant catalytic stability in the broad range of pH from 6.0 to 8.0 was noted. A sharp decrease in the enzyme activity rate was detected at acidic pHs, at pH 5.0 the enzyme retained about 59% of its initial activity. Similarly, pH stability of L-methioninase from *A. ustus* [2], *A. favipes* [24] and *T. harzianum* [12] had maximum stability in the pH range from 6.5 to 8.5. [14] recorded that, *C. tropicalis* L-methioninase kept its activity at pH 5.5 up to pH 7.5, a slight decrease in the enzyme activity was observed at pH 8.0 while at pH 5.0 the enzyme retained about 81% of its initial activity. The rapid loss of enzymatic stability at acidic pHs may be caused by protein denaturation or dissociation of pyridoxal-5-phosphate or unfolding of the enzyme active site [6, 12]. Interestingly, the pH stability range of *A. fumigatus* L-methioninase is close to the blood pH (7.4) ensuring its therapeutic value [6].

### Substrate specificity of *A. fumigatus* L-methioninase and kinetic parameters

**Fig. 5:**
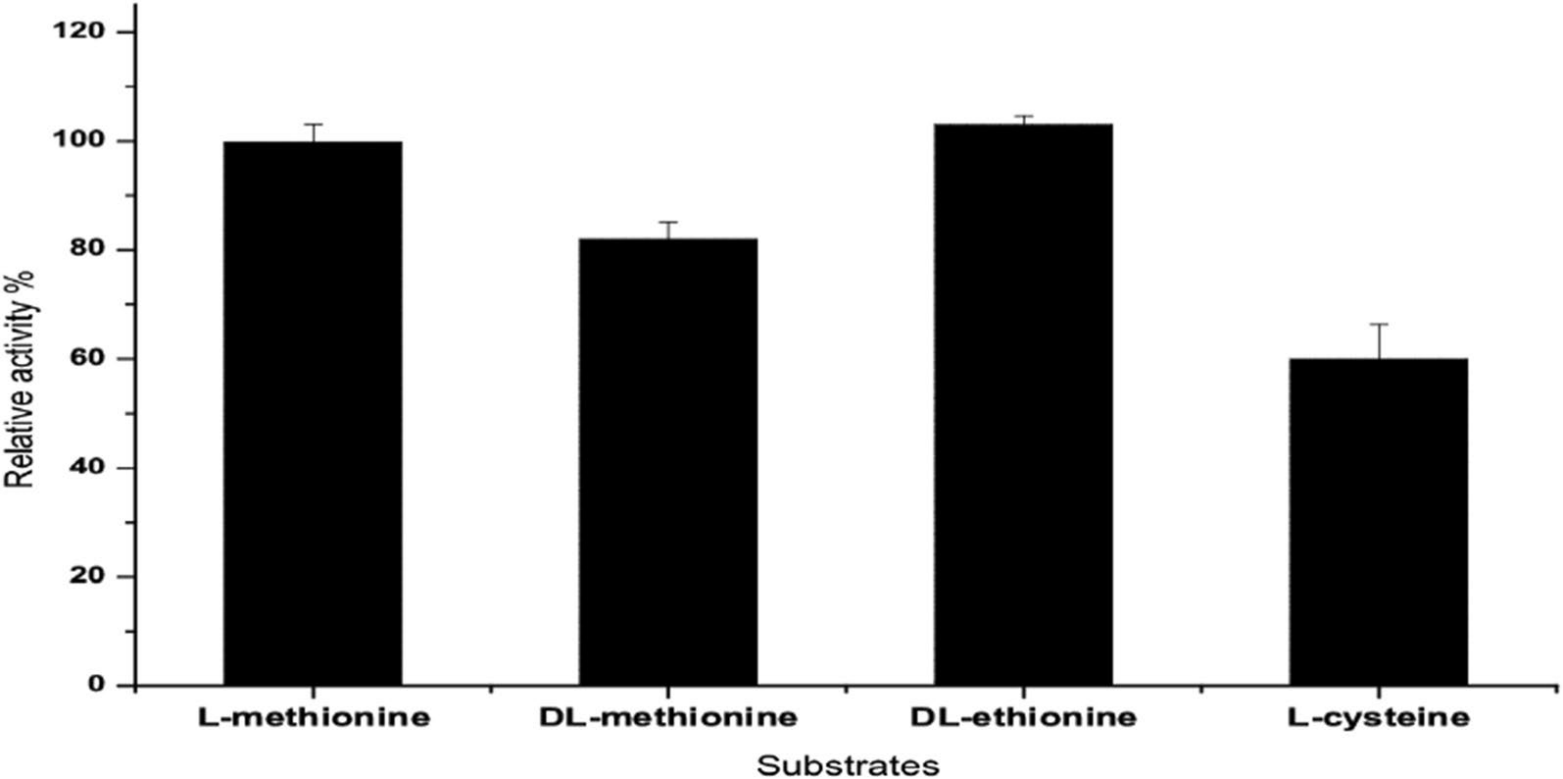
Substrate specificity of *A. fumigatus* L-methioninase

This experiment was designed to evaluate the specificity of *A. fumigatus* L-methioninase towards various substrates. Equal amount (20 mM) of standard substrate L-methionine and its related substrates, DL-methionine, DL-ethionine, and L-cysteine were added separately to the reaction mixture and incubated under optimum assay conditions. Data obtained from (Fig. 5) showed varied enzymatic relative activities against these substrates. *A. fumigatus* L-methioninase had lower efficiency to degrade DL-methionine (82%), and L-cysteine (60%) when compared to the standard substrate, L-methionine. Meanwhile, the performance of *A. fumigatus* L-methioninase against L-ethionine was found, higher than L-methionine with 3%. Indeed, *A. fumigatus* L-methioninase had a distinctly affinity toward the standard substrate and its analog, DL-ethionine as reported by many authors [6, 27, 28]. In agreement with our results, *A. flavipes* L-methioninase [24], and *T. harzianum* L-methioninase [12] had relative catalytic activity towards different amino acids; high specificity was found against L-methionine followed by L-cystine and L-cysteine. Substrate specificity of *A. ustus* L-methioninase showed the highest relative activity against the standard substrate only, and the enzyme had a lower relative activity against other substrates [2].

The multifunctional activity of L-methioninase for hydrolyzing the C-S and C-O rather than the C–C bonds is mostly observed for bacterial [29–32], protozoal [33, 34], and fungal enzymes [24, 35]. Studies on the substrate specificity of *C. tropicalis* L-methioninase showed that the enzyme had a relative activity towards different sulfur containing amino acids [14].

**Fig. 6:**
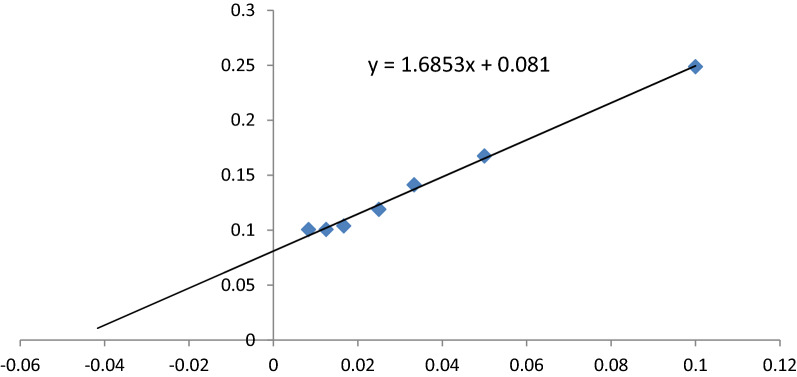
Lineweaver–Burk Plot of *A. fumigatus* L-methioninase against L-methionine

Otherwise, the kinetic parameters such as Michalis-Menten constant (Km) and maximum velocity (Vmax) of *A. fumigatus* L-methioninase were calculated by incubating the enzyme with various concentrations of the standard substrate, L-methionine (10–120 mm) under optimized assay conditions. The apparent Km and Vmax was calculated from the Lineweaver–Burk plot relating 1/V to 1/[S] (Fig. 6).

From Linweaver-Burk plot we can summarized that, higher affinity and maximum velocity of L-methioninase toward L-methionine was obtained with 20.608 mM and 12.34568 µM.min^−1^, respectively. The enzyme showed maximal activity at a substrate level of 40, 60, and 80 mM for L-methionine, which indicates that the active center of the enzyme became saturated with its substrate at concentrations above 30 mM. The effect of substrate concentrations on the velocity of the reaction showed hyperbolic rather than sigmoid relationship with L-methioninase towards its substrates indicating this enzyme may be classified as allosteric enzyme. The apparent K_m_ of L-methioninase was indicating high affinity of the enzyme to its substrate reflecting its potential efficiency as antitumor agent. Concerning the catalytic efficiency, the fungal L-methioninase was more than that observed for bacterial ones as clearly obtained in the values of K_m_ for L-methionine. L-methioninase from *Clostridium sporogenes* it have minimal specificity to L-methionine as a substrate (K_m_ value 90 mM), indicating its low therapeutic value [29]. While L-methioninase from *A. flavipes* [24], *T. harzianum* [12], and *A. ustus* [2] exhibited greater affinity to L-methionine, which may afford the enzyme clinically.

### Effect of metal ions and inhibitors on *A. fumigatus* L-methioninase

*A. fumigatus* L-methioninase activity was pre-incubating with different metal ions and chemical reagents at two concentrations 10 mM and 1 mM respectively, for 4 h at room temperature. Following that, the enzyme solution was added to its substrate and the residual activity was determined by the standard assay method.

The results obtained were tabulated in (Table 2), from which it is noteworthy that most of the metal salts not significantly contributed in the enzyme activation. Conversely, a considerable decrease of L-methioninase activity, more than 40% was noted with Hg^2+^, Cu^2+^, Li^2+^ and Mn^2+^. In addition, the slight inhibition of enzymatic activity was recorded with Ni^2+^, Fe^3+^, Cr^3+^. As evident from these results, *A. fumigatus* L-methioninase did not need any mineral ions for its catalytic activity. On the other hand, the metal chelating agent EDTA had insignificantly effect, which indicated the non-metallic nature of L-methioninase. The effect of sodium dodecyl sulfate (SDS) as a strong surfactant and sodium azide on the enzyme was remaining 40.5% of its activity, while thiol reducing agents such as β- mercabtoethano, and DTT inhibited the enzyme activity and retaining only about 62.5% and 86% of its original activity respectively. Therefore, *A. fumigatus* L-methioninase contain reactive –SH group in the active site center and suggests the existence of disulfde bond for preserving the molecular structure of enzyme [6, 12] Furthermore, Inhibition of *A. fumigatus* L-methioninase activity in the presence of Cu^2+^ might be ensure the presence of the sulfhydryl group in the active site of the enzyme [14]. In this finding, the activity of L-methioninase from *A. flavipes* was highly inhibited by Hg2^+^, Cu2^+^ and Fe^2+^ [24]. In addition, [14] reported that, L-methioninase from *C. tropicalis* was completely inhibited by Cd + 2 and Cu + 2 while it was enhanced by Na^+^, Ni^2+^ and Mg^2+^. In accordance with our findings, Metal ions such as Ca^2+^, Mg^2+^, Zn^2+^ and Cd^2+^ showed slight inhibition of *T. harzianum* L-methioninase activity [12]. In addition, the insensitivity of *A. fumigatus* L-methioninase to organic solvent such as methanol and ethanol makes it an excellent candidate for utilization in biotechnological sectors [23].

**Table 2:** Effect of different metal ions and chemical reagents on *A. fumigatus* L-methioninase

Metal ions (Chloride salt, 10 mM)	Relative activity (%)	Chemical reagents (1 mM)	Relative activity (%)
Na^+1^	100 ± 1.5	Sodium Azide	42 ± 1
K^+1^	100 ± 0.88	8-Hydroxyquinoline	91.3 ± 1.5
Mg^+2^	100 ± 1	EDTA	100 ± 1.1
Mn^+2^	55.5 ± 1.7	SDS	52 ± 1
Zn^+2^	93 ± 2.1	DTT	86 ± 0.88
Ca^+2^	92.8 ± 1.1	β- mercabtoethanol	62.5 ± 1.3
Ba^+2^	98 ± 1.5	Ethanol	97.3 ± 0.81
Li^+2^	52.7 ± 1.3	Methanol	100.7 ± 1.5
Cu^+2^	49.2 ± 1	n-Butanol	86 ± 1
Co^+2^	100 ± 1.6		
Cd^+2^	70 ± 2.9		
Cr^+3^	88 ± 0.84		
Ni^+2^	84.9 ± 1.6		
Fe^+3^	78.9 ± 1.4		
Hg^+2^	29.8 ± 1.7		

### Effect of *A. fumigatus* L-methioninase on cancer and normal cell lines In-vitro

**Fig. 7:**
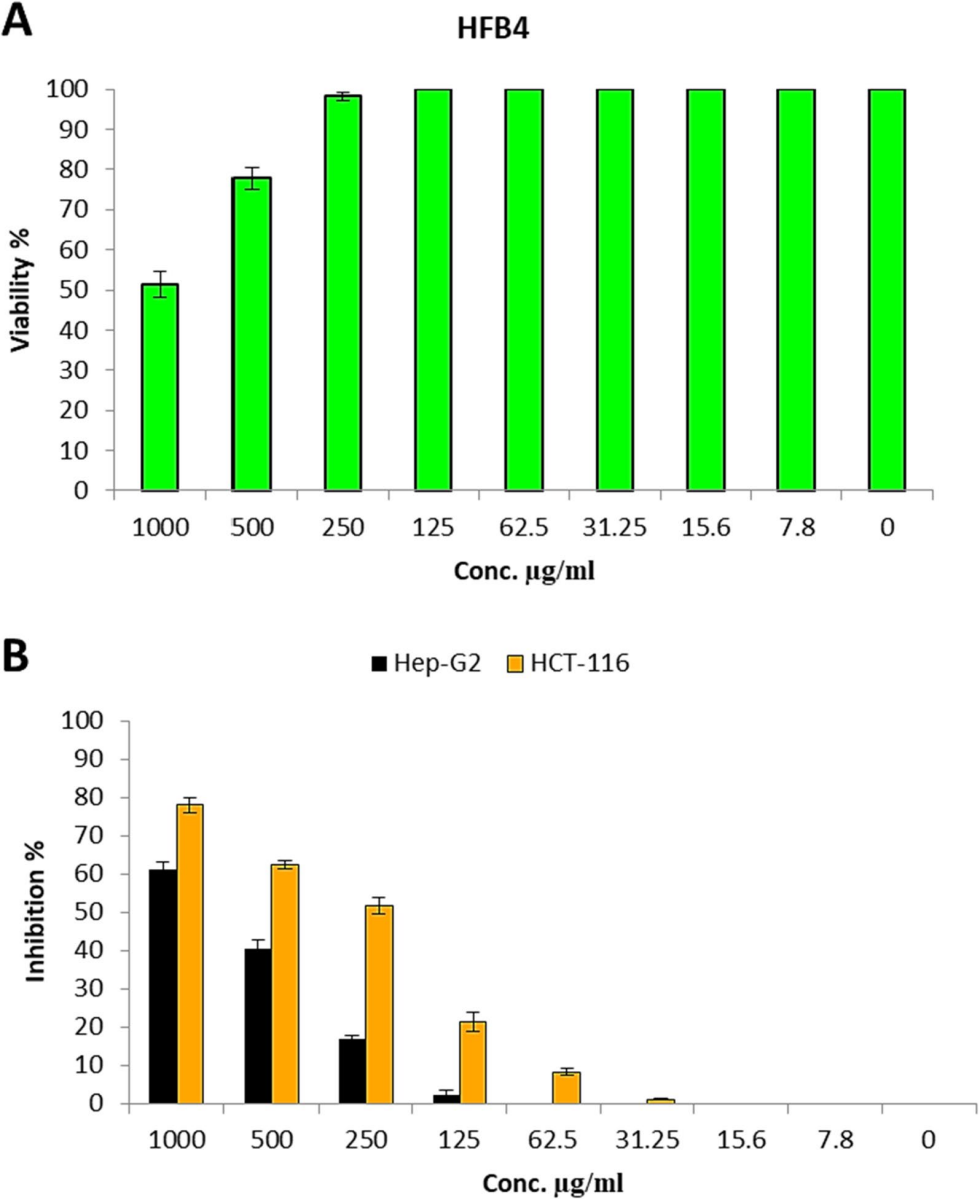
Anticancer and Cytotoxicity of *A. fumigatus* L-methioninase

The elevated dependence of tumor cells on the essential amino acid; methionine is a cancer-specific metabolic defect [6, 36]. In-vitro evaluation of *A. fumigatus* L-methioninase against two cancer cell line, human Hepatocellular carcinoma cells (HEPG2), and human Colon carcinoma cells (HCT-116) was performed with different doses as shown in Fig. 7B. Cytotoxicity of *A. fumigatus* L-methioninase was also investigated against Human normal melanocytes cells (HFB4) with different concentrations (Fig. 7A), *A. fumigatus* L-methioninase had an efficacy against both cancer cell lines and this effect was a concentration dependent. Since, a remarkable anticancer activity of L-methioninase against cancer cells was found to be potent had IC_50_ (Concentration which resulted in 50% inhibition of cell growth) values less than 1 Unite as appeared in case of colon HCT116; 243 ± 4.87 µg/ml (0.486 U/ml), and in case of liver HepG-2; 726 ± 29.31 µg/ml (1.452 U/ml). In contrast, the results revealed that L-methioninase had a much higher IC_50_ more than 1000 µg/ml (< 2 U/ml) and this indicate that, the toxicity of L-methioninase toward the growth of normal melanocytes HFB4 cells was negligible when compared to the activity against cancer cells.

As reported previously, *A. fumigatus* L-methioninase showed anticancer specificity preferable than other fungal L-methioninase. In this way, *T. harzianum* L-methioninase showed an anticancer activity against Hep-G2 cell lines followed by MCF-7 cell lines with inhibition ratio of 47.62 and 33.84%, respectively at I U/mL [12]. El-Sayed et al. [37] showed that, the plausible antiproliferative activity of purified L-methioninase from *A. flavipes* towards the five experimented cell lines, and this effect was a concentration dependent. The enzyme has a strong efficiency against prostate (IC_50_, 0.001 U/ml), liver (IC_50_, 0.269 U/ml), breast (IC_50_, 0.372 U/ml), lung (IC_50_, 2.0 U/ml) and colon (IC_50_, 2.6 U/ml). Anticancer activity of the purified enzyme was evaluated in-vitro using different cancer and normal cell line. The observed results generally indicated that, the activity of L-methioninase against cancer cell lines was highly effectiveness while the cytotoxic effect toward normal cells was negligible. Our results corroborate the earlier findings of Tan et al. [36], they treated twenty-one different human cancer cell lines and seven human normal cell lines in-vitro with recombinant MGL. They found that high cytotoxic activity of MGL towards cancer cell lines compared to the normal cell lines and suggested the cells from different kinds of cancer are methionine dependent which can also affect their DNA methylation. The exact mode of MGL anticancer is fully understood by [36, 38] who suggested that, the amount of methionine needed by the cancer cells is much greater than that required by the normal cells, this may be related to the increased protein synthesis and enhanced trans-methylation reactions. This ensures that multiple biochemical reactions necessary for the fast proliferation of cancer cells and have notably action on DNA lead to sharp damage of cell membrane which undergoing apoptosis resulted in the death of the cells.

## Conclusion

In the current study, L-methioninase from thermo-tolerant fungus *A. fumigatus* was purified with 3.04-fold and 73.9% of enzymatic recovery. The enzymatic biochemical properties showed a maximum activity at pH 7, 30–40 °C and the enzymatic stability was noted up to 40 °C. Additionally, L-methioninase had potential anticancer activity against Hep-G2 and HCT-116 cancerous cell lines without any toxicity on normal cell line. Eventually, L-methioninase from thermo-tolerant fungus *A. fumigatus* is a promising candidate as anticancer agent which can be used in the medical therapy.

## Data Availability

Not applicable.
