# Correction to: purification, characterization and anticancer activity of L-methionine γ-lyase from thermo-tolerant aspergillus fumigatus

**DOI:** 10.1186/s12934-023-02062-w

**Published:** 2023-03-25

**Authors:** Mahmoud H. Hendy, Amr H. Hashem, Waleed B. Suleiman, Mahmoud H. Sultan, Mohamed Abdelraof

**Affiliations:** 1 Botany and Microbiology Department, Faculty of Science, Al-Azhar University, Cairo, 11884 Egypt; 2 Microbial Chemistry Department, National Research Centre, 12622 Dokki, Cairo, Egypt

**Correction to: Microbial Cell Factories (2023) 22:8**

10.1186/s12934-023-02019-z

In the original publication of the article, the third author name Waleed B. Suleiman was incorrectly published as “Waleed B. Sulieman”. The original article has been corrected.

